# Novel PDMS-b-PPO Membranes Modified with Graphene Oxide for Efficient Pervaporation Ethanol Dehydration

**DOI:** 10.3390/membranes12090832

**Published:** 2022-08-25

**Authors:** Mariia Dmitrenko, Anastasia Chepeleva, Vladislav Liamin, Anna Kuzminova, Anton Mazur, Konstantin Semenov, Anastasia Penkova

**Affiliations:** 1 St. Petersburg State University, 7/9 Universitetskaya nab., St. Petersburg 199034, Russia; 2 Pavlov First Saint Petersburg State Medical University, L’va Tolstogo Ulitsa 6–8, St. Petersburg 197022, Russia

**Keywords:** membrane, poly(2,6-dimethyl-1,4-phenylene oxide), polydimethylsiloxane, block copolymer, graphene oxide, pervaporation, ethanol dehydration

## Abstract

Purification and concentration of bioalcohols is gaining new status due to their use as a promising alternative liquid biofuel. In this work, novel high-performance asymmetric membranes based on a block copolymer (BCP) synthesized from polydimethylsiloxane (PDMS) and poly(2,6-dimethyl-1,4-phenylene oxide) (PPO) were developed for enhanced pervaporation dehydration of ethanol. Improvement in dehydration performance was achieved by obtaining BCP membranes with a “non-perforated” porous structure and through surface and bulk modifications with graphene oxide (GO). Formation of the BCP was confirmed by Fourier-transform infrared (FTIR) and nuclear magnetic resonance (NMR) spectroscopies. The changes to morphology and physicochemical properties of the developed BCP and BCP/GO membranes were studied by scanning electron (SEM) and atomic force (AFM) microscopies, thermogravimetric analysis (TGA) and contact angle measurements. Transport properties of the developed membranes were evaluated by the pervaporation dehydration of ethanol over a wide concentration range (4.4–70 wt.% water) at 22 °C. The BCP (PDMS:PPO:2,4-diisocyanatotoluene = 41:58:1 wt.% composition) membrane modified with 0.7 wt.% GO demonstrated optimal transport characteristics: 80–90 g/(m^2^h) permeation flux with high selectivity (76.8–98.8 wt.% water in the permeate, separation factor of 72–34) and pervaporation separation index (PSI) of 5.5–2.9.

## 1. Introduction

Currently, the growth of populations and development of industrial sectors leads to the active use of energy [1]. The reserves of petroleum, fossil fuels and other traditional sources of energy are irretrievably depleted over time. This causes active development of environmentally friendly and cost-effective fuel alternatives. Biofuels are a promising newcomer among energy resources despite production problems, and they are gaining position as promising products in the fuel and energy complex [2,3]. Liquid biofuels such as bioalcohols produced by microbial fermentation require intensive purification and concentration to ensure purity [4]. The most important stage affecting the production cost and the quality of biofuels is the dehydration of bioalcohols. Traditional methods for this (for example, azeotropic and extractive distillation, absorption, solvent extraction and adsorption) have significant limitations such as high energy consumption, high costs, low efficiency and difficulty in scaling [1]. This leads to the development of alternative methods for dehydrating bioalcohols, such as membrane processes [5,6], which are related to sustainable processes due to their advantages in environmental friendliness, economy, high selectivity, energy efficiency and ease of automation [7,8]. Pervaporation is considered one of the most effective membrane methods for the separation of liquid mixtures of low-molecular-weight substances, in particular azeotropic ones, especially with the use of mixed matrix membranes [2,9,10,11,12]. Pervaporation with the correct membrane could replace the alcohol concentration step required for reconstitution [13,14].

For the preparation of membranes for dehydration, the most commonly used polymers are polyvinyl alcohol [15,16], chitosan [17,18,19], alginate [20,21,22], polyacrylonitrile [23,24], polyamides [25,26], etc. Among these polymeric membrane materials, an aromatic glassy polymer poly(2,6-dimethyl-1,4-phenylene oxide) (PPO) also deserves attention due to its characteristics (chemical resistance and high mechanical strength) [27,28], and PPO membranes are selective to water in vacuum pervaporation [29,30]. However, PPO has been investigated in only a few studies for the pervaporation dehydration of ethanol [31,32,33].

Comparison of pervaporation performance of dense PPO and sulfonated polyphenylene oxide (SPPO) membranes in the separation of water/ethanol mixtures was carried out in [31]. The PPO membrane demonstrated a good separation factor (~150–10) but low permeation flux (210–140 g/(m^2^h)) in pervaporation dehydration of ethanol (10–90 wt.% water) at 25 °C. To improve the permeability, the PPO membrane was sulfonated (SPPO). The SPPO membrane had improved permeation flux of 300 g/(m^2^h) and a separation factor up to 700 in the pervaporation dehydration of ethanol (10 wt.% water) at 25 °C due to increased hydrophilicity caused by the sulfonation. Hollow carbon-fiber membranes derived from SPPO were developed and evaluated for the dehydration of alcohols (methanol, ethanol, 2-propanol and 1-butanol) at 50–80° C [32]. This membrane demonstrated water flux of 0.24 kg/(m^2^h), ethanol flux of 0.0059 kg/(m^2^h) and a separation factor of 360 in the separation of water/ethanol (10/90 wt.%) mixture at 60 °C. In [33], pervaporation dense PPO membranes modified with 1 and 2 wt.% fullerene (C_60_) were developed for ethanol dehydration. The fullerene-modified membrane had increased permeability and maintained high selectivity with respect to water compared to the pristine PPO membrane. The PPO/C_60_ (2 wt.%) membrane had optimal transport characteristics for pervaporation dehydration of ethanol (10–70 wt.% water): ~70–98 wt.% water in the permeate and normalized permeation flux of 1.1–0.7 kg·µm/(m^2^h). Thus, for the promising use of pervaporation PPO membranes in ethanol dehydration, highly efficient membranes are required.

In the present work, to significantly improve dehydration performance, the PPO and polydimethylsiloxane (PDMS) were considered good candidates to be combined by copolymerization for the preparation of pervaporation asymmetric membranes. This approach has already shown its relevance [34] and effectiveness in [14], where effective alcohol-selective pervaporation membranes based on polydimethylsiloxane-block-polyphenylene oxide (PDMS-b-PPO) copolymer synthesized by a bridge reagent technique were developed. The PDMS-b-PPO copolymer was prepared using PPO with average molecular weight of 16 000 g/mol and PDMS dual-terminated with an aminopropyl group. The formation of membranes was carried out with the use of n-butyl alcohol and chloroform as solvents for casting solutions. This led to uniform dispersion of PDMS domains in the PPO matrix and outer skin layer of prepared membranes, resulting in the formation of membranes highly selective to ethanol in pervaporation. The PDMS-b-PPO membrane had 3.8 kg/(m^2^h) permeation flux and a separation factor of 8.53 in pervaporation separation of ethanol/water (5/95 wt.%) mixture at 60 °C. In this work, another poly(2,6-dimethyl-1,4-phenylene oxide) and hydroxy-terminated PDMS were used for the preparation of a block copolymer to develop water-selective membranes. It should also be noted that only chloroform was applied as a solvent for the casting solutions.

It is also worth noting that earlier in [33] it had already been shown that modification of the PPO membrane with a carbon particle–fullerene is promising. In the present work, improvement of developed membranes based on the synthesized PDMS-b-PPO copolymer was carried out by modification with graphene oxide (GO). The introduction of GO into a membrane significantly changes the morphology of the polymer matrix and the functionalized membrane surface, affecting the hydrophilic–hydrophobic properties, which leads to significant improvement of transport properties in pervaporation dehydration of ethanol [30,35]. To the best of our knowledge, there are no works on the use of mixed matrix PDMS-b-PPO copolymer membranes modified with GO in pervaporation.

Thus, in the present work, novel high-performance mixed matrix membranes based on a block copolymer (BCP) synthesized from PDMS and PPO and modified with GO were developed to enhance pervaporation dehydration of ethanol. Significant improvement in performance was achieved by obtaining asymmetric BCP membranes with a “non-perforated” porous structure via a non-solvent-induced phase separation (NIPS) technique, varying the PDMS-b-PPO copolymer composition to obtain the optimal properties and modification with GO to further improve permeability and selectivity of BCP membranes. The structural and physicochemical properties of the developed BCP-based membranes were studied by Fourier-transform infrared (FTIR) and nuclear magnetic resonance (NMR) spectroscopies, scanning electron (SEM) and atomic force (AFM) microscopies, thermogravimetric analysis (TGA) and contact angle measurements. Transport properties of the developed membranes were compared with those of the dense PPO membrane and evaluated in pervaporation dehydration of ethanol over a wide concentration range.

## 2. Materials and Methods

### 2.1. Materials

Poly(2,6-dimethyl-1,4-phenylene oxide) (PPO, 1.06 g/mL at 25 °C, “Sigma-Aldrich”, St. Petersburg, Russia) and polydimethylsiloxane (PDMS, hydroxy terminated, 0.94 g/mL at 25 °C, “Sigma-Aldrich”, St. Petersburg, Russia) were applied for synthesis of a PDMS-b-PPO copolymer, which was used as a membrane matrix. Graphene oxide (GO, “Fullerene Technologies”, St. Petersburg, Russia) [36] was used as a modifier of the PDMS-b-PPO membranes. 2,4-diisocyanatotoluene (TDI, 95 wt.%) used as a cross-linking agent and dibutyltin dilaurate (DBTDL, 95 wt.%) used as a catalyst were purchased from “Sigma-Aldrich” (St. Petersburg, Russia). Chloroform (CHCl_3_, 99.1 wt.%), chlorobenzene (C_6_H_5_Cl, 99.7 wt.%), methanol (MeOH, 99.5 wt.%) and ethanol (EtOH) purchased from “Vecton” (St. Petersburg, Russia) were used without additional treatment.

### 2.2. Synthesis of the PDMS-b-PPO Copolymer

**Figure 1:**
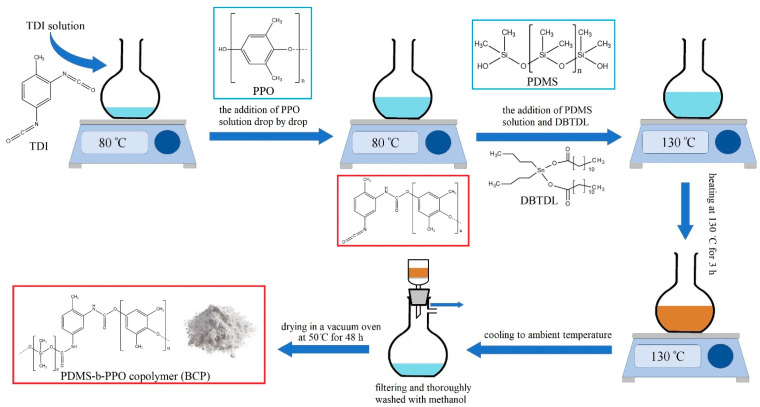
The scheme of a block copolymer (BCP) preparation.

The determined amounts of PDMS, PPO and TDI were dissolved in chlorobenzene under constant stirring to obtain 10 wt.% solutions. First, the TDI solution was poured into a reactor flask and heated to 80 °C. The PPO solution was added drop-by-drop to the TDI solution with intensive stirring at 80 °C in order for one of the isocyano (–NCO) groups of TDI to react with a hydroxyl (-OH) group of PPO to form urethane, and also for the inhibition of TDI polymerization [3]. Then, a mixture of the PDMS solution and DBTDL (3 mL) were added to the system, where PDMS hydroxyl groups should attack the other isocyano (-NCO) groups of TDI. After that, the temperature was raised gradually to 130 °C, where it was maintained for 3 h to complete the reaction. The sediment (block copolymer) was formed after cooling the system to ambient temperature. The block copolymer (BCP) was filtered, thoroughly washed with methanol, and dried in a vacuum oven at 50 °C for 48 h [1]. Block copolymers with a different ratios of components (PDMS:PPO:TDI) were synthesized (26:73:1, 41:58:1, 51:48:1 and 71:28:1 wt.%). The scheme of BCP preparation is presented in Figure 1 [14].

### 2.3. Membrane Preparation

Preparation of the membranes based on BCP was carried out via a non-solvent-induced phase separation (NIPS) technique. A precalculated amount of BCP to obtain 18 wt.% solution was dissolved in chloroform under stirring for 2 h at ambient temperature, with subsequent ultrasonic treatment for 1 h at ambient temperature to remove gases from the polymer solution [1]. To obtain asymmetric membranes, the BCP solution was cast on a glass support using a casting blade (with gap width of 200 μm), which was further immersed in a coagulation bath with methanol at 25 °C. The obtained membrane was removed from the glass plate and dried in vacuum oven for 20 h. The BCP membrane with the PDMS:PPO:TDI ratio of 71:28:1 wt.% was impossible to form by NIPS due to a large amount of PDMS in BCP composition. PDMS cannot be directly converted to an asymmetric membrane by phase inversion due to its poor mechanical properties. The thickness of the BCP membranes was measured by a micrometer to be 35 ± 5 µm.

To compare with the BCP-based membranes, a dense PPO membrane was also prepared according to the following procedure: a pre-determined amount of polymer to obtain 8 wt.% PPO solution was dissolved in chloroform under stirring for 3 h with ultrasonic treatment for 30 min [30]. Then, the PPO solution was poured onto cellophane fixed on a hollow steel ring, followed by solvent evaporation in an oven at 40 °C for 12 h and then separation of the formed membrane from the cellophane [30]. The thickness of the dense PPO membrane was measured by a micrometer to be 35 ± 5 µm.

**Figure 2:**
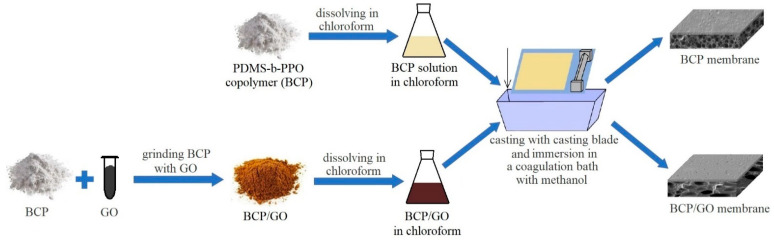
Preparation scheme of the BCP-based membranes.

Preparation of the BCP/GO composites was carried out by solid-phase synthesis [2]: the determined amount of BCP was ground with a calculated amount of GO (0.3, 0.5, 0.7 or 0.9 wt.% with respect to the BCP weight). The BCP/GO membranes were prepared according to the technique described above for unmodified BCP membranes. The preparation scheme of the BCP and BCP/GO membranes is demonstrated in Figure 2.

### 2.4. Pervaporation

**Figure 3:**
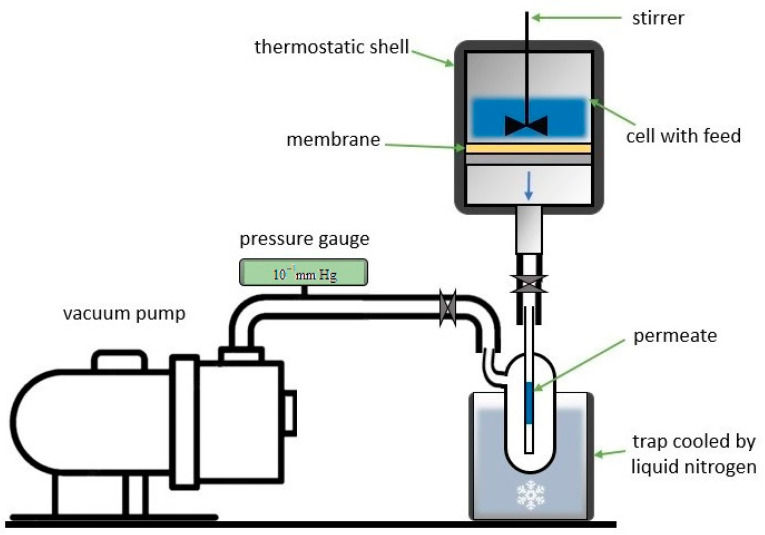
Schematic of pervaporation setup.

Transport properties of the dense PPO and asymmetric BCP-based membranes were investigated for pervaporation dehydration of ethanol over a wide concentration range (4.4–70 wt.% water) at 22 °C in a steady-state stirring cell with an effective membrane area of 9.6 cm^2^ and <10^−1^ mm Hg downstream pressure [9,37]. The pervaporation setup schematic is demonstrated in Figure 3.

BCP-based membrane performance is presented in terms of permeation flux, water content in the permeate, component permeability, separation factor, membrane selectivity and pervaporation separation index. All these calculated parameters are additionally presented in Tables S1 and S2 in the Supplementary Materials. Permeation flux (*J*) was calculated as follows [38]:

(1) $$J=\frac{W}{A\times t},$$

where *W* is the permeate weight (kg), *A* is the effective membrane area (m^2^), and *t* is the permeate collection time (h).

Component permeability ($P_{i}^{G}$), commonly reported in Barrers (1 Barrer = 1 × 10^−10^ cm^3^ (STP) cm/cm^2^ s cmHg), was calculated as follows [39]:

(2) $$P_{i}^{G}=j_{i}\frac{l}{p_{i_{0}}-p_{i_{l}}},$$

where $j_{i}$ is partial flux, *l* is membrane thickness, and $p_{i_{0}}$ and $p_{i_{l}}$ are the partial vapor pressures of component *i* on either side of the membrane.

Membrane selectivity (*α*), defined as the ratio of component permeabilities, was calculated as follows [39]:

(3) $$\alpha =\frac{P_{i}^{G}}{P_{J}^{G}},$$

The separation factor (*β*) was calculated as follows [39]:

(4) β=yiyjxixj,

where *y_i_* and *y_j_* are the weights of components *i* and *j*, respectively, in the permeate; *x_i_* and *x_j_* are the weights of components *i* and *j*, respectively, in the feed. As the values of membrane selectivity were comparatively similar to those of the separation factor, they are presented in Tables S1 and S2 in the Supplementary Materials.

The pervaporation separation index (PSI) was calculated as follows:

(5) PSI=J×(β−1)

The permeate and feed compositions were analyzed and controlled by gas chromatography using a Chromatek Crystal 5000.2 chromatograph (“Chromatec”, Nizhny Novgorod, Russia) with a “Hayesep R” column. Pervaporation experiments for each membrane were carried out at least three times, after which the average values of the parameters were calculated and taken for analysis. The mean accuracy of transport parameters of the BCP-based membranes was ±0.5% for water content in the permeate and ±15% for the permeation flux.

### 2.5. Fourier-Transform Infrared Spectroscopy (FTIR)

The structure of the BCP membranes was studied using an IRAffinity-1S spectrometer (“Shimadzu”, St. Petersburg, Russia) over the range of 500–4000 cm^−1^ and with an attenuated total reflectance (ATR) accessory (“PIKE Technologies”, St. Petersburg, Russia) at 25 °C.

### 2.6. Nuclear Magnetic Resonance (NMR)

The BCP membranes were investigated using a Bruker Avance III 400 WB NMR spectrometer (“Bruker”, Bremen, Germany) with a magnetic field of 9.4 T and a CP/MAS probe of 4 mm. Nuclei ^13^C Larmor frequency was 100.64 MHz, and an external reference for ^13^C nuclei was liquid tetramethylsilane (TMS).

### 2.7. Scanning Electron Microscopy (SEM)

The morphology of BCP-based membranes was studied using a Zeiss Merlin SEM (“Carl Zeiss SMT”, Oberhochen, Germany). To prevent surface modification and charging, the investigation was carried out at the low electron beam current of 100 pA and accelerating voltage of 1 kV.

### 2.8. Atomic Force Microscopy (AFM)

The surface topography of the BCP-based membranes was studied using an NT-MDT NTegra Maximus atomic force microscope (“NT-MDT Spectrum Instruments”, Moscow, Russia) in the tapping mode.

### 2.9. Contact Angle Measurements

To evaluate the hydrophilic–hydrophobic surface balance of the BCP-based membranes, contact angles were measured by the sessile drop method using a Goniometer LK-1 (“NPK Open Science” Ltd., Krasnogorsk, Russia). The “DropShape” software was used to calculate and analyze results.

### 2.10. Thermogravimetric Analysis (TGA)

The thermochemical properties of the BCP-based membranes were studied using a Thermobalance TG 209 F1 Libra (“Netzsch”, Leuna, Germany) in a temperature range of 30–950 °C and under an Ar atmosphere.

## 3. Results and Discussion

### 3.1. Transport Properties of the BCP-Based Membranes

**Figure 4:**
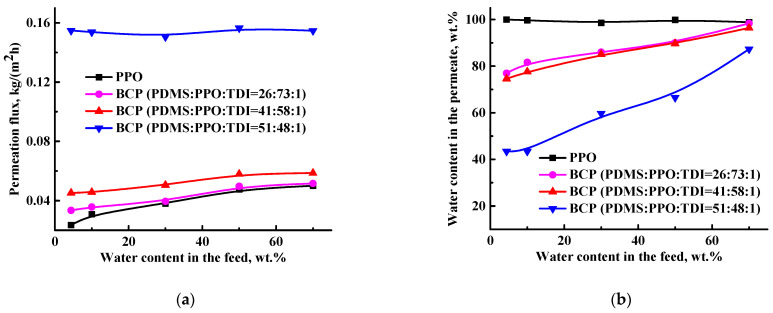

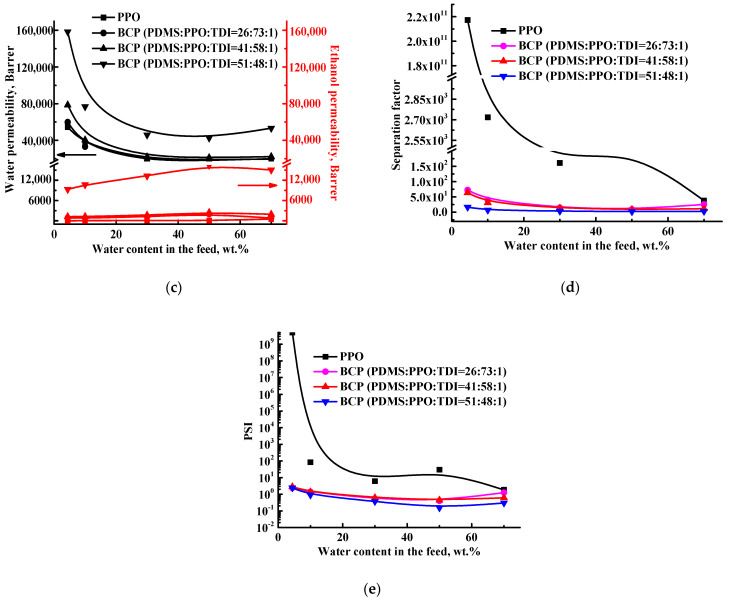
The dependence of (**a**) permeation flux, (**b**) water content in the permeate, (**c**) component permeability, (**d**) separation factor and (**e**) PSI on water content in the feed for the PPO and BCP-based membranes in pervaporation dehydration of ethanol (4.4–70 wt.% water) at 22 °C.

The transport properties of the developed BCP-based membranes were evaluated for ethanol dehydration by pervaporation. The choice of the water–alcohol system was due to the importance of absolute ethanol as an industrial chemical in various industries (pharmaceutical, chemical, medical, etc.) and as a promising alternative fuel for automobiles. Separation of a water/ethanol mixture by traditional separation methods is complicated due to the formation of an azeotrope with a water content of 4.4 wt.% [40]. This is energetically and economically unfavorable and requires the addition of toxic intermediate agents to form stronger azeotropic mixtures with water, which makes alcohols unsuitable for use in the pharmaceutical and food industries, where a high purity degree is required. This separation problem is easily solved by environmentally friendly pervaporation using the developed membranes. Membranes based on the BCP with different ratios of the components PDMS:PPO:TDI (26:73:1, 41:58:1 and 51:48:1 wt.%) were tested for pervaporation dehydration of ethanol over a wide concentration range (4.4–70 wt.% water) (Figure 4). Transport properties of the dense PPO membrane were also estimated to compare with the BCP-based membranes.

Membranes based on PPO are known to transmit small molecules (for example, water) and to be selective for water in vacuum pervaporation [29,30]. The mechanism of mass transfer through PPO membranes may be described as follows: an organic substance interacts with PPO due to higher solubility and swelling, forming bonds and creating transport channels for water. Therefore, as the BCP contains a greater extent of PPO (73, 58 or 48 wt.%), membranes based on it have been demonstrated to transmit water (Figure 4b). We have shown that for the BCP-based membranes, permeation flux increased and water content in the permeate decreased (Figure 4a,b) with increasing PDMS concentration. It should be noted that the permeation flux of the BCP-based membranes was significantly higher than that of the dense PPO membrane with a decrease in water in the permeate. This performance could be explained by morphology changes (the formation of a “non-perforated” structure with sponge cross-section organization because of the NIPS preparation technique, confirmed by SEM data, presented below) and increased surface roughness (confirmed by AFM data, presented below) of the BCP-based membranes [6]. The decrease of water content in the permeate was conditioned by significantly increasing the permeability of the BCP-based membranes, which induced joint penetration of ethanol with water. The developed BCP membrane with PDMS:PPO:TDI = 51:48:1 composition had the highest permeation flux (~0.2 kg/(m^2^h)) but the lowest water content in the permeate (43.5–87.3 wt.%). These changes are associated with a high content of PDMS in the composition of the BCP, which contributes to a more porous membrane structure, causing an increase in permeability and a significant decrease in selective properties.

Based on these transport parameters, component permeability (component flux normalized for membrane thickness and driving force), separation factor (β) and pervaporation separation index (PSI) were calculated and presented in Figure 4. It was demonstrated that permeation flux (Figure 4a) was dominated by water flux, namely, the water permeability for all membranes was higher than the ethanol permeability [41] (Figure 4c). We found that water permeability decreased, in contrast to permeation flux, and ethanol permeability slightly increased for membranes with increased water content in the feed. The BCP membrane with PDMS:PPO:TDI = 51:48:1 composition had the highest values of water permeability, which is in agreement with the highest permeation flux (Figure 4a). Further, this membrane had the highest values of ethanol permeability and the lowest selectivity.

Figure 4d,e show the plots of the separation factor and PSI as a function of water concentration in the feed: both parameters for the BCP-based membranes slightly fall with increased water content in the feed. It should be mentioned that for the PPO membrane, these changes were more significant and higher in terms of values compared to BCP membranes. This was caused by the high water content in the permeate (more than 98.6 wt.%) for the PPO membrane, which made a significant contribution to the increased separation factor and PSI values.

The developed BCP membrane with PDMS:PPO:TDI = 41:58:1 composition had the optimal transport properties in pervaporation dehydration of ethanol (4.4–70 wt.% water): ~2 times increased permeation flux (0.05–0.06 kg/(m^2^h)) with a slight decrease in selective properties (74.6–96.4 wt.% water in the permeate, 73–26 separation factor) and 2.8–0.6 PSI compared to the dense PPO membrane (0.02–0.05 kg/(m^2^h), 99.9–98.8 wt.% water in the permeate, 2·10^11^–38 separation factor and 5.1·10^9^–1.8 PSI).

**Figure 5:**
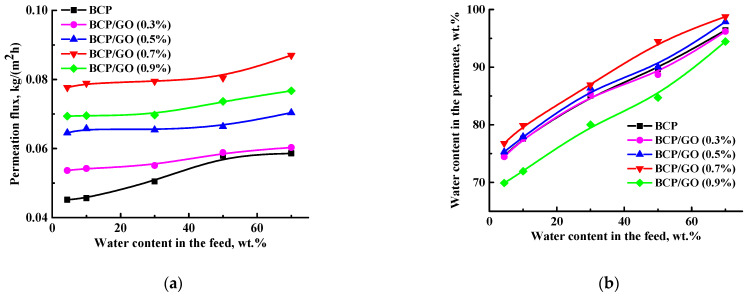

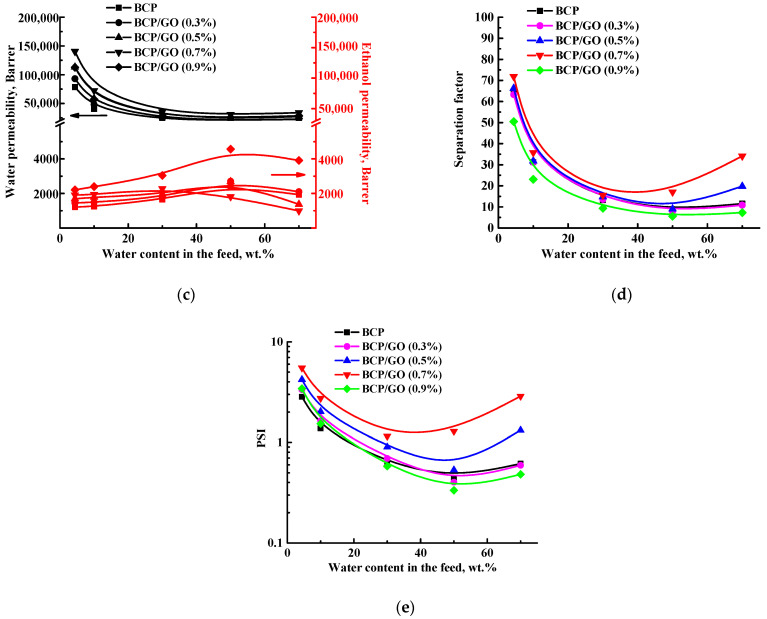
The dependence of (**a**) permeation flux and (**b**) water content in the permeate, (**c**) component permeability, (**d**) separation factor and (**e**) PSI on water content in the feed for the BCP and BCP/GO membranes in pervaporation dehydration of ethanol (4.4–70 wt.% water) at 22 °C.

For further improvement of transport performance, the BCP (with PDMS:PPO:TDI = 41:58:1 composition) membrane was modified with GO nanoparticles (0.3, 0.5, 0.7 or 0.9 wt.%). The modified BCP/GO membranes were also tested in pervaporation dehydration of ethanol over a wide concentration range (4.4–70 wt.% water) (Figure 5). Transport properties of the pristine BCP membrane are also repeated in Figure 5 to compare with the GO-modified membranes.

Introduction of GO into the BCP matrix led to increased permeation flux and water content in the permeate (Figure 5a,b), and increased water permeability, separation factor and PSI (Figure 5c–e) with increasing GO concentration in the membrane matrix, except for the BCP/GO (0.9%) membrane. The BCP/GO (0.9%) membrane demonstrated lower permeation flux (Figure 5a) compared to the BCP/GO (0.7%) membrane, the lowest water content in the permeate, and decreased separation factor and PSI (Figure 5b,d,e). This could be due to agglomeration of the GO nanoparticles in the BCP matrix hindering mass transfer of the feed components through the membrane [7]. Further, agglomeration of GO particles led to the formation of larger pores in the membrane structure (confirmed by SEM data, presented below), contributing to joint penetration of ethanol and water, causing the decrease in selectivity. This is also confirmed by this membrane having the highest ethanol permeability values (Figure 5c).

The increased permeation flux and water content in the permeate of the modified BCP membranes could be explained by changes to the internal and surface structures: a dense sponge structure with “non-perforated” pores and surface hydrophilization after GO-modification were confirmed with SEM and contact angle data, presented below. The BCP/GO (0.7%) membrane demonstrated the highest values of the transport characteristics in pervaporation dehydration of ethanol (4.4–70 wt.% water): ~2 times higher permeation flux (0.08–0.09 kg/(m^2^h)), a high level of selectivity (76.8–98.8 wt.% water in the permeate and separation factor of 72–34), the highest water permeability (140503–33880 Barrer) and 5.5–2.9 PSI compared to the pristine BCP (PDMS:PPO:TDI = 41:58:1) membrane: 0.04–0.06 kg/(m^2^h), 74.6–96.4 wt.% water in the permeate, separation factor of 64–12, water permeability of 78639–22286 Barrer and 2.8–0.6 PSI. Thus, BCP/GO (0.7%) membrane was demonstrated to be promising for industrial ethanol dehydration.

### 3.2. Characterization of the BCP-Based Membranes

**Figure 6:**
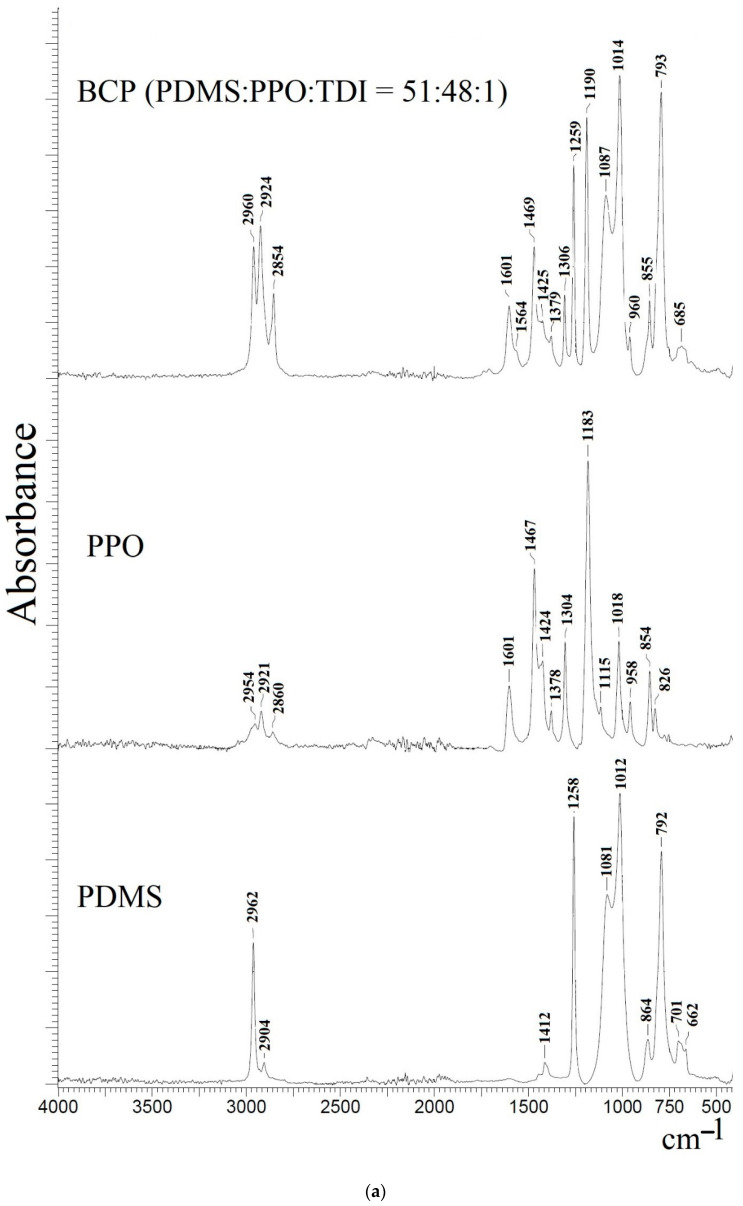

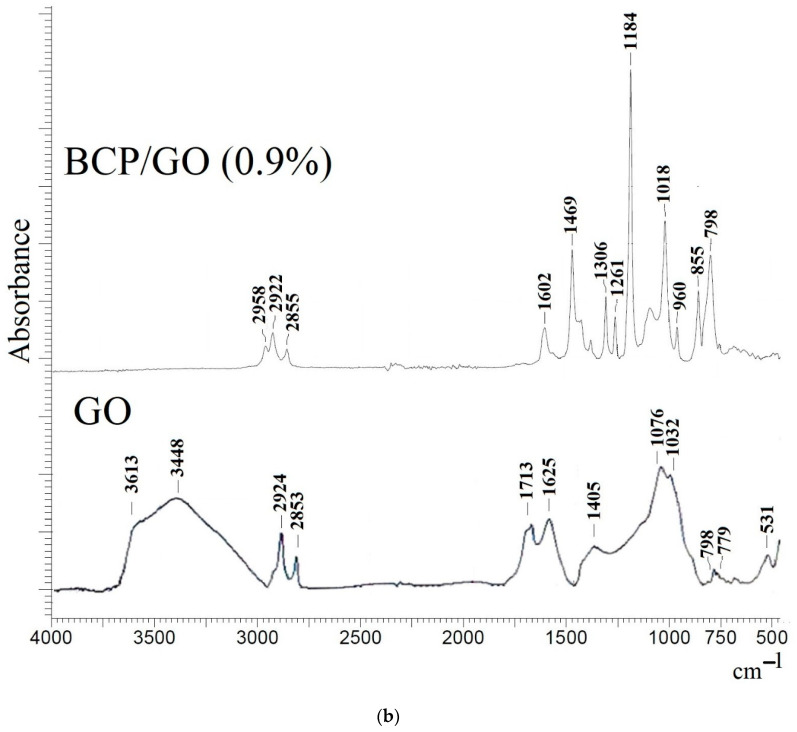
FTIR spectra of (**a**) the PDMS and PPO polymers and BCP (PDMS:PPO:TDI = 51:48:1) membrane and (**b**) GO powder and BCP (PDMS:PPO:TDI = 41:58:1)/GO (0.9%) membrane.

The PPO and PDMS polymers as well as GO, BCP (PDMS:PPO:TDI = 51:48:1) and BCP (PDMS:PPO:TDI = 41:58:1)/GO (0.9%) membranes were studied using FTIR spectroscopy to confirm the formation of the block-copolymer and to study structural changes (Figure 6).

The PDMS spectrum demonstrates absorption peaks at 2962 and 2904 cm^−1^ corresponding to the stretching vibrations for CH_3_ (C–H bond), at 1258 cm^−1^ related to the typical absorption for a Si–CH_3_ bond and at 1100–1000 cm^−1^ assigned to asymmetric stretching vibration for Si–O–Si [8]. The PPO spectrum shows peaks at 2954, 1601 and 1467 cm^−1^ referring to the vibrations of the benzene rings, at 1183 and 1304 cm^−1^ corresponding to symmetric and asymmetric vibrations of C–O bonds and at 2921, 2860 and 854 cm^−1^ assigned to C–H bond vibrations [7,9]. In the BCP membrane spectrum, the characteristic peaks of both PPO and PDMS polymers are found. Furthermore, the peak at 1712 cm^−1^ was discovered for the BCP membrane, indicating the formation of the urethane groups (C=O bond) of the formed PDMS-b-PPO copolymer [10].

Modification of the BCP (PDMS:PPO:TDI = 41:58:1) membrane with 0.9 wt.% GO did not lead to significant changes to peak position or intensity. In the modifier spectrum, characteristic peaks of GO were observed: at 3448 cm^−1^, the wide peak corresponding to the -OH stretching of the carboxylic group; at 1713 cm^−1^, the peak related to the C=O vibration of the carboxylic groups; and at 1625 cm^−1^, the peak attributed to the C=C of the graphene aromatic domain [42,43]. These peaks were not visible in the spectrum of the BCP (PDMS:PPO:TDI = 41:58:1)/GO (0.9%) membrane due to low GO concentration in the membrane. This may indicate weak interactions or hydrogen bonding between the polymer and the GO particles.

**Figure 7:**
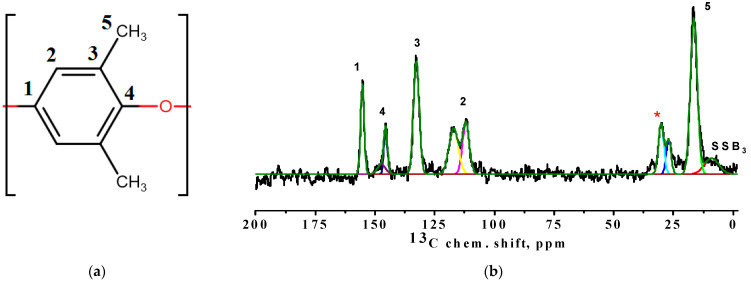
(**a**) Schematic representation of a PPO monomer with numbered nonequivalent carbon atoms (1, 2, 3, 4, and 5) and (**b**) ^13^C NMR spectrum of the BCP membrane. Asterisk refers to the group of peaks at about 30 ppm corresponding to the methyl groups of PDMS.

The formation of the block copolymer was also confirmed using NMR spectroscopy. Figure 7 demonstrates a schematic representation of a PPO monomer with numbered nonequivalent carbon atoms and the ^13^C NMR spectrum of the BCP membrane.

**Figure 8:**
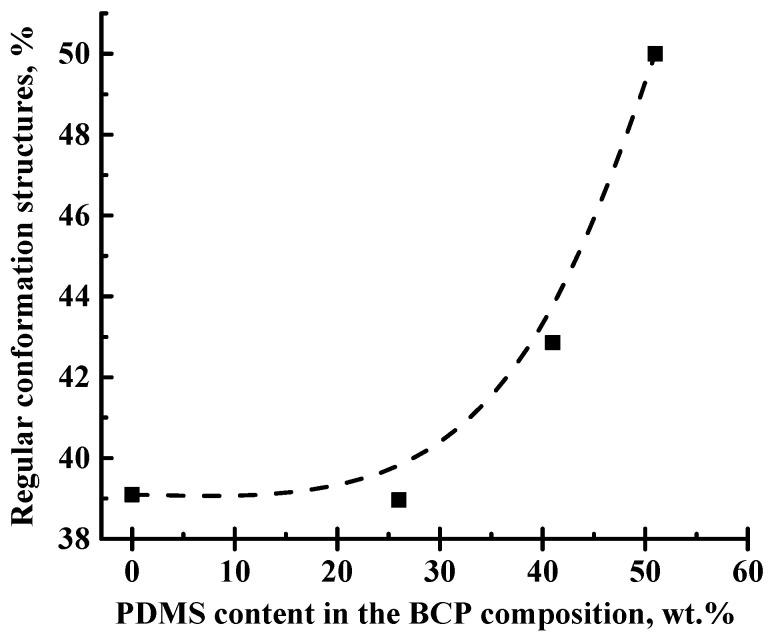
The dependence of regular conformation structure proportion on the PDMS content in the BCP composition.

There are six resolved peaks in the ^13^C NMR spectrum, five of which (numbered from 1 to 5 on the spectrum) correspond to carbon atoms in different non-equivalent positions of PPO (Figure 7a). A weak peak next to the line that corresponds to the methyl groups of the PPO is a satellite rotation (SSB) from the line at the 3 position of the polymer unit (Figure 7b). A group of peaks at about 30 ppm corresponds to the methyl groups of PDMS (asterisk in Figure 7b) [11]. In addition, the spectral line corresponding to the carbon atoms in the 4 position of the polymer unit is divided into two weakly resolved components. This may be due to the presence of regions with a regular conformational packing of polymer units in the membrane structure [44]. Since the component, which is about 112 ppm, has a smaller width, it can be assumed that it corresponds to the conformationally ordered polymer phase [13]. Estimating the ratio of the integral areas of these two considered components of the spectrum, it is possible to evaluate the change in the content of regular conformation structures in the samples (Figure 8). It was demonstrated that the increase in PDMS concentration in the BCP composition led to the rise of the regular conformation structure proportion, which largely caused the increase in the permeability of the BCP membranes with an increase in PDMS content.

**Figure 9:**
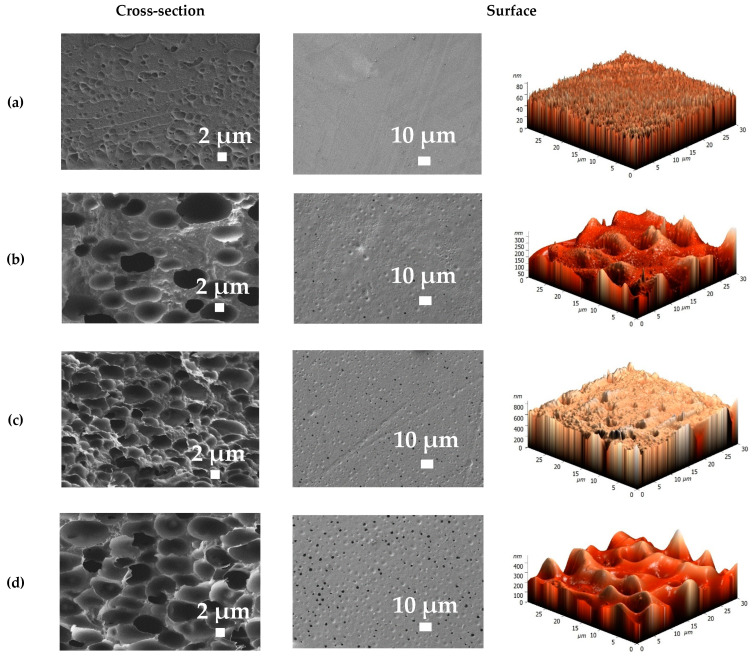
Cross-sectional and surface SEM micrographs and AFM images of the (**a**) PPO and BCP (**b**) PDMS:PPO:TDI = 26:73:1, (**c**) PDMS:PPO:TDI = 41:58:1 and (**d**) PDMS:PPO:TDI = 51:48:1 membranes.

**Figure 10:**
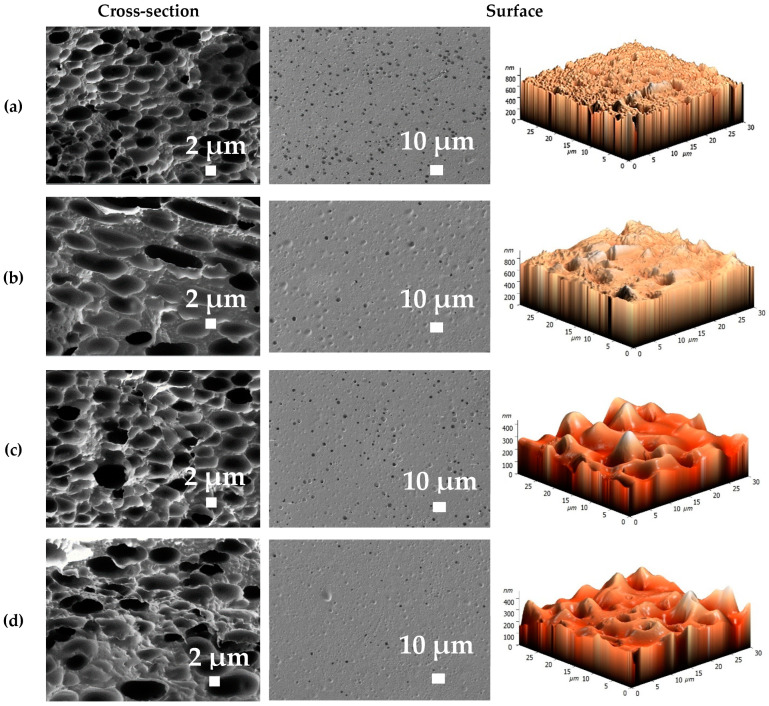
Cross-sectional and surface SEM micrographs and AFM images of the (**a**) BCP/GO (0.3%), (**b**) BCP/GO (0.5%), (**c**) BCP/GO (0.7%) and (**d**) BCP/GO (0.9%) membranes.

The morphology and surface topography of the developed membranes based on PPO, BCP and BCP/GO composites were studied by SEM and AFM (Figure 9 and Figure 10). It is known that PDMS tends to not form an asymmetric membrane by phase inversion due to poor mechanical properties. However, the BCP membranes have a holistic homogeneous structure with “non-perforated” pores due to the presence of more rigid PPO segments (Figure 9). The casting solution consisting of BCP dissolved in chloroform was deposited onto a glass support and immersed in a coagulation bath with methanol, and phase inversion occurred due to solvent displacement between the liquid component and the gel, resulting in the formation of a BCP membrane with a porous structure [14,45].

The presented SEM micrographs of the unmodified PPO membrane obtained by solvent evaporation demonstrate a dense homogeneous structure of the surface and cross-section with uniform plastic deformations (Figure 9a). Synthesis of the BCP and the formation of the BCP membranes by NIPS led to the formation of a spongy cross-section and porous membrane surface, increasing permeability of the BCP membranes compared to the PPO membrane (Figure 4). However, the pores of the BCP membranes were “non-perforated” and non-hollow. This effect was also confirmed in [1], where the new PDMS-b-PPO copolymer membranes with high flux for alcohol permselective pervaporation were developed. The newly formed morphology of the BCP membranes is mainly the result of specific hard and soft segments of polymers and the membrane formation process (by NIPS). Further, an increase of the PDMS concentration in the BCP solution (from 26 to 41 wt.%) led to the formation of membranes with a tighter spongy cross-section and the formation of more surface pores (Figure 9b,c). Increasing PDMS content to 51 wt.% in the BCP contributed to the formation of a membrane with a large-porous cross-section and larger pores on the surface (Figure 9d), since the high content of PDMS leads to a higher degree of microphase separation [1]. This change caused a significant increase in the performance of the BCP membrane prepared from the copolymer with the PDMS:PPO:TDI = 51:48:1 composition and a decrease of water content in the permeate (Figure 4). 

The introduction of GO into the BCP matrix (PDMS:PPO:TDI = 41:58:1) leads to the formation of membranes with fewer pores in the cross-section and surface and changes their shapes (more oblong and larger) (Figure 10). This effect intensified with increasing the GO concentration from 0.3 to 0.9 wt.% in the BCP membrane. Changes in morphology increased productivity and selectivity of the modified BCP/GO membranes (Figure 5). 

Based on the AFM images, all membranes had a typical nodule structure [30]. Further, the AFM data confirmed the surface SEM micrographs, where introduction of GO into the BCP matrix resulted in higher roughness due to the formation of more pores on the surface. Using AFM images (Figure 9 and Figure 10), the surface roughness parameters in terms of average roughness (Ra) and root-mean-squared roughness (Rq) were calculated (Table 1). To assess the change in hydrophilic–hydrophobic surface properties of membranes after modification, contact angles of water were measured, with the results also presented in Table 1.

The BCP membranes had a greater surface roughness compared to the dense PPO membrane, which intensified with increasing PDMS in the composition of the BCP. This is because the method of membrane formation and pore formation on the surface created a larger effective contact area of the BCP membranes with the separated mixture during pervaporation, increasing productivity. The introduction of GO into the BCP matrix did not significantly change the roughness of the membrane surface (less than 4 nm difference) compared to the BCP (PDMS:PPO:TDI = 41:58:1) membrane. With an increase of the PDMS content in the BCP, the surface becomes hydrophobic: the contact angle of the BCP membranes increases compared to that of the PPO membrane. Modification of the BCP (PDMS:PPO:TDI = 41:58:1) membrane by GO led to surface hydrophilization (the decrease in the contact angle from 95 to 92°) due to functional (oxygen-containing) groups of GO that migrated to the membrane surface and improved the hydrophilic properties, causing improvement in permeation flux and water selectivity for the modified membranes (Figure 5) [46,47]. It should be noted that the BCP-based membranes with high contact angle values (more than 90°) were selective with respect to water in pervaporation dehydration of ethanol. This may be explained as follows: as the BCP contains a greater percentage of PPO (73, 58 or 48 wt.%), the BCP-based membranes demonstrate the characteristic transport properties for PPO’s ability to transmit water and to be selective for water in vacuum pervaporation [29,33,48]. This specific mechanism of water mass transfer through PPO membranes can be described as follows: due to higher solubility (according to Hansen’s solubility parameters) and swelling, organic substances interact with PPO, forming bonds and creating transport channels for water penetration [30]. Membranes with water contact angles greater than 90° having enhanced pervaporation dehydration has also been confirmed in [29,33,48,49]. The mechanisms of water molecule penetration can be different depending on the nature of the polymer (diffusive selectivity due to strong polymer packing, sorption selectivity due to specific interactions of penetrants with membrane materials, etc.).

**Figure 11:**
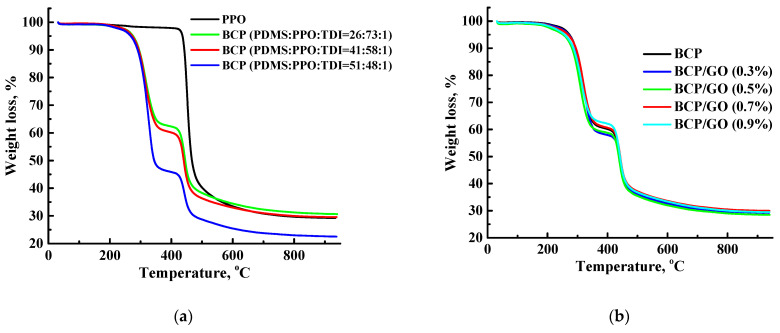
Thermogravimetric (TG) curves for the (**a**) PPO and BCP and (**b**) BCP and BCP/GO membranes.

Thermochemical properties and stability of the membranes based on PPO, BCP and BCP/GO were studied by thermogravimetric analysis (Figure 11).

The TG curve of the PPO membrane demonstrates two stages of weight loss: (1) until 450 °C, weight loss is associated with the evaporation of the residual solvent and low-molecular-weight impurities [14]; (2) after 450 °C, weight loss is due to the degradation of polymer chains (Figure 11a) [7]. In the BCP membranes, there was one more stage of weight loss from 370 °C to 450 °C, associated with depolymerization of PDMS in BCP [15]. The greatest weight loss was observed for the BCP (PDMS:PPO:TDI = 51:48:1) membrane, which had the highest content of PDMS in the BCP composition. The introduction of GO into the BCP (PDMS:PPO:TDI = 41:58:1) matrix did not lead to significant changes to the thermochemical properties of the modified membranes (Figure 11b), which is consistent with previous work [16]. Thus, it was shown that the developed membranes based on BCP and its composite with GO were thermally stable over a wide temperature range, which is promising for industrial applications at elevated temperatures.

### 3.3. Comparison of Membrane Performance in the Pervaporation Dehydration of Ethanol

The performance of the developed BCP/GO (0.7%) membrane for the pervaporation separation of the azeotropic water/ethanol (4.4/95.6 wt.%) mixture was compared with the literature-described polymeric membranes in terms of permeation flux and separation factor at comparable experiment conditions (Table 2).

The PPO/GO (0.7%) membrane had higher permeation flux in pervaporation dehydration of ethanol (4.4 wt.% water) compared to the PVA and Alg-based membranes developed in [50,54,56], but it was largely inferior in selective properties (low separation factor of 72). Compared to the PVA/PVP/PMA (4%) and NaAlg/HPA (6%) membranes [51,52], the membrane developed in this study had slightly lower permeation flux with a higher separation factor. The P-CS and P-SA membranes [53,55] had significantly superior transport properties (permeation flux and separation factor) compared to the BCP/GO (0.7%) membrane. However, it should be mentioned that the P-CS membrane performed well at low water concentrations in the feed and had decreased selective properties with the water content increasing from 10.23 to 52.3 wt.% [53]. The P-SA membrane was tested in pervaporation dehydration of ethanol up to 38.6 wt.% water [55], at which point the separation factor decreased from 2182 to 6.58. The BCP/GO (0.7%) membrane was evaluated in dehydration up to 70 wt.% water with an increasing separation factor.

The transport properties of the developed BCP/GO (0.7%) membrane in the pervaporation dehydration of ethanol (10 wt.% water) were also compared with the fullerene-modified PPO membranes [33] (Table 3).

The BCP/GO (0.7%) membrane had lower permeation flux during separation at ambient temperature compared to PPO and PPO/C_60_ membranes, but it possessed higher selective properties (separation factor of 36). The permeation flux of this developed membrane can be increased by the use of higher pervaporation temperatures, while the high thermal stability of this membrane was confirmed by TGA (Figure 11). Thus, the BCP/GO (0.7%) membrane had the optimal transport characteristics in pervaporation dehydration of ethanol compared with polymeric membranes described in the literature, and it is promising for industrial alcohol dehydration.

## 4. Conclusions

In this study, novel pervaporation high-performance mixed matrix membranes based on the synthesized PDMS-b-PPO copolymer modified with GO were developed for improved ethanol dehydration.

Firstly, to enhance the transport characteristics of PPO polymer for ethanol dehydration, membranes based on BCP with different ratios of PPO and PDMS were developed. These membranes had significantly higher permeation flux with lower water content in the permeate compared to the dense PPO membrane in pervaporation dehydration of ethanol over a wide concentration range (4.4–70 wt.% water). This can be explained by morphology changes to the BCP membranes: the formation of an asymmetric “non-perforated” structure with a sponge cross-section organization due to NIPS (confirmed by SEM) and increased surface roughness (confirmed by AFM). Formation of the PDMS-b-PPO copolymer was confirmed by FTIR and NMR spectroscopies. The developed BCP membrane with PDMS:PPO:TDI = 41:58:1 composition had optimal transport properties in pervaporation dehydration of ethanol (4.4–70 wt.% water): ~2 times increased permeation flux (0.05–0.06 kg/(m^2^h)) with decreased selective properties (74.6–96.4 wt.% water in the permeate, 64–12 separation factor) and a decreased pervaporation separation index of 2.8–0.6 compared to the dense PPO membrane.

Further, to improve the transport properties of the developed BCP membrane, it was modified with GO. The introduction of GO (0.3–0.9 wt.%) into the BCP matrix increased both permeation flux and water content in the permeate in pervaporation dehydration of ethanol (4.4–70 wt.% water). This was due to internal and surface structural changes during modification: the formation of a dense sponge structure and surface hydrophilization (confirmed by SEM and contact angle data). The BCP/GO (0.7%) membrane demonstrated the highest values for the transport characteristics: ~2 times higher permeation flux (0.08–0.09 kg/(m^2^h)), the highest selectivity (76.8–98.8 wt.% water in the permeate, separation factor of 72–34) and pervaporation separation index (PSI) of 5.5–2.9 compared to the pristine BCP membrane. Thus, the asymmetric BCP/GO (0.7%) membrane is highly efficient and promising for industrial ethanol dehydration.

## Figures and Tables

**Table 1:** Surface roughness parameters and contact angles of water of the dense PPO, BCP and BCP/GO membranes.

Membrane	Surface Parameters		Contact Angle of Water, °
	Ra, nm	Rq, nm	
PPO	4.0	5.2	89 ± 2
BCP (PDMS:PPO:TDI = 26:73:1)	28.1	38.0	91 ± 2
BCP (PDMS:PPO:TDI = 41:58:1)	33.0	58.0	95 ± 2
BCP (PDMS:PPO:TDI = 51:48:1)	41.6	64.9	98 ± 2
BCP/GO (0.3%)	33.4	52.5	95 ± 2
BCP/GO (0.5%)	37.3	58.9	94 ± 2
BCP/GO (0.7%)	35.5	61.8	93 ± 2
BCP/GO (0.9%)	34.0	53.2	92 ± 2

**Table 2:** Comparison of transport properties for the membranes in the pervaporation separation of azeotropic water/ethanol (4.4/95.6 wt.%) mixture and mixtures close to this composition.

Membrane	Feed-Water Content, wt.%	Temperature, °C	Permeation Flux, g/(m^2^h)	Separation Factor (*β*)	Reference
BCP/GO (0.7%)	4.4	22	79	72	This study
PVA/TEOS	4	RT	30	375	[50]
PVA/TEOS/STA (5%)	4	RT	40	2949	
PVA/TEOS/STA (10%)	4	RT	52	5377	
PVA/TEOS/STA (15%)	4	RT	67	8622	
PVA/PVP/PMA (4%)	4	27	100	10	[51]
NaAlg/HPA (6%)	4	30	170	60	[52]
P-CS	3.5	30	250	670	[53]
Alg/DNA-Ca^2+^	3.5	40	10	5500	[54]
Alg/DNA-Mg^2+^	3.5	40	10	6500	
P-SA	5.2	30	240	2182	[55]
PVA	6.25	45	22	1143	[56]
PVA/PES	6.25	45	33	950	

PVA, polyvinyl alcohol; TEOS, tetraethyl orthosilicate; PES, polyethersulfone; RT, room temperature; STA, silicotungstic acid nanoparticle; P-CS, phosphorylated chitosan; PVP, polyvinyl pyrrolidone; PMA, phosphomolybdic acid; P-SA, phosphorylated sodium alginate; NaAlg, sodium alginate; DNA, deoxyribonucleate; HPA, Preyssler type heteropolyacid H_14_[NaP_5_W_30_O_110_].

**Table 3:** Comparison of transport properties of the PPO- and BCP-based membranes in the pervaporation dehydration of ethanol (10 wt.%).

Membrane	Temperature, °C	Permeation Flux, g/(m^2^h)	Separation Factor (*β*)	Reference
BCP/GO (0.7%)	22	79	36	This study
PPO	50	700	13	[33]
PPO/C_60_ (1%)	50	920	16	
PPO/C_60_ (2%)	50	1100	21	

## Data Availability

Data is contained within these article and supplementary materials.

## Supplementary Materials

The following supporting information can be downloaded at: https://www.mdpi.com/article/10.3390/membranes12090832/s1, Table S1: Transport parameters (permeation flux, water content in the permeate, component (water and ethanol) permeability, separation factor (β), membrane selectivity (α), and pervaporation separation index (PSI)) for the PPO and BCP-based membranes in pervaporation dehydration of ethanol (4.4–70 wt.% water) at 22°C.; Table S2: Transport parameters (permeation flux, water content in the permeate, component (water and ethanol) permeability, separation factor (β), membrane selectivity (α), and pervaporation separation index (PSI)) for BCP (PDMS:PPO:TDI = 41:58:1 wt.%) and BCP/GO membranes in pervaporation dehydration of ethanol (4.4–70 wt.% water) at 22°C.
