# Supplementary material for: Novel PDMS-b-PPO Membranes Modified with Graphene Oxide for Efficient Pervaporation Ethanol Dehydration
Source: Membranes (Basel). 2022 Aug 25;12(9):832. doi: 10.3390/membranes12090832 (PMC9505798; doi:10.3390/membranes12090832)
Supplement: Supplementary file 1 [file membranes-12-00832-s001.zip › membranes-1866223-supplementary.pdf]

SUPPLEMENTARY MATERIALS

Table S1. Transport parameters (permeation flux, water content in the permeate, component (water and ethanol) permeability, separation factor (β), membrane selectivity (α), and pervaporation separation index (PSI)) for the PPO and BCP-based membranes in pervaporation dehydration of ethanol (4.4-70 wt.% water) at 22°C.

Transport parameter	Membrane / Water content in feed, wt.%	PPO	BCP (PDMS:PPO:TDI 26:73:1)	BCP (PDMS:PPO:TDI 41:58:1)	BCP (PDMS:PPO:TDI 51:48:1)
Permeation flux, kg/(m^2h)	4.4	0.023	0.033	0.045	0.155
	10	0.031	0.036	0.046	0.154
	30	0.038	0.040	0.050	0.151
	50	0.048	0.050	0.058	0.156
	70	0.050	0.052	0.059	0.155
Water content in permeate, wt.%	4.4	100	77.0	74.6	43.5
	10	99.7	81.7	77.6	43.5
	30	98.6	86.1	85.1	59.7
	50	99.8	90.1	89.7	66.5
	70	98.9	98.4	96.4	87.3

Continuation of Table S1. Transport parameters (permeation flux, water content in the permeate, component (water and ethanol) permeability, separation factor (β), membrane selectivity (α), and pervaporation separation index (PSI)) for the PPO and BCP-based membranes in pervaporation dehydration of ethanol (4.4-70 wt.% water) at 22°C.

Transport parameter	Membrane / Water content	PPO	BCP (PDMS:PPO:TDI 26:73:1)	BCP (PDMS:PPO:TDI 41:58:1)	BCP (PDMS:PPO:TDI 51:48:1)

	in feed, wt.%				
Water permeability, Barrer	4.4	5451	59854	78639	158384
	10	35219	33235	40448	76795
	30	19202	17452	22006	45940
	50	19255	18175	21167	42610
	70	19453	19953	22286	53242
Ethanol permeability, Barrer	4.4	$2\cdot10^{-7}$	815	1217	9260
	10	13	799	1250	10655
	30	119	1209	1651	13264
	50	31	1995	2428	16422
	70	513	779	1932	15030
Separation factor (β)	4.4	$2\cdot10^{11}$	73	64	17
	10	2718	40	31	7
	30	161	14	13	3
	50	624	9	9	2
	70	38	26	12	3
Membrane selectivity (α)	4.4	$2\cdot10^{11}$	73	65	17
	10	2817	42	32	7
	30	161	14	13	3
	50	625	9	9	2
	70	38	26	12	3

Continuation of Table S1. Transport parameters (permeation flux, water content in the permeate, component (water and ethanol) permeability, separation factor (β), membrane selectivity (α), and pervaporation separation index (PSI)) for the PPO and BCP-based membranes in pervaporation dehydration of ethanol (4.4-70 wt.% water) at 22°C.

**Transport**	**Membrane** / **Water**	**PPO**	**BCP (PDMS:PPO:TDI 26:73:1)**	**BCP (PDMS:PPO:TDI 41:58:1)**	**BCP (PDMS:PPO:TDI 51:48:1)**

	**content in feed, wt.%**				
**PSI**	4.4	$5.1\cdot10^{9}$	2.4	2.8	2.4
	10	83.6	1.4	1.4	0.9
	30	6.1	0.5	0.6	0.4
	50	29.6	0.4	0.4	0.2
	70	1.8	1.3	0.6	0.3

Table S2. Transport parameters (permeation flux, water content in the permeate, component (water and ethanol) permeability, separation factor (β), membrane selectivity (α), and pervaporation separation index (PSI)) for BCP (PDMS:PPO:TDI = 41:58:1 wt.%) and BCP/GO membranes in pervaporation dehydration of ethanol (4.4-70 wt.% water) at 22°C.

**Transport parameter**	**Membrane** / **Water content in feed, wt.%**	**BCP**	**BCP/GO (0.3%)**	**BCP/GO (0.5%)**	**BCP/GO (0.7%)**	**BCP/GO (0.9%)**
**Permeation flux, kg/(m^2h)**	4.4	0.045	0.054	0.065	0.078	0.069
	10	0.046	0.054	0.066	0.079	0.070
	30	0.050	0.055	0.065	0.079	0.070
	50	0.058	0.059	0.066	0.080	0.074
	70	0.059	0.060	0.070	0.087	0.077
**Water content in permeate, wt.%**	4.4	74.6	74.5	75.3	76.8	69.9
	10	77.6	77.6	77.9	79.9	71.9
	30	85.1	85.4	86.4	86.9	80.0
	50	89.7	88.7	90.0	94.5	84.7
	70	96.4	96.2	97.9	98.8	94.4
**Water permeability, Barrer**	4.4	78639	93082	113302	140503	112798
	10	40448	48193	58689	72471	57260
	30	22006	24122	28958	35396	28493
	50	21167	21271	24331	30922	25366
	70	22286	22829	27199	33880	28566
**Ethanol permeability, Barrer**	4.4	1217	1453	1690	1909	2209
	10	1250	1491	1782	1944	2394
	30	1651	1770	1956	2275	3042
	50	2428	2704	2689	1810	4569
	70	1932	2099	1371	990	3915

Continuation of Table S2. Transport parameters (permeation flux, water content in the permeate, component (water and ethanol) permeability, separation factor (β), membrane selectivity (α), and pervaporation separation index (PSI)) for BCP (PDMS:PPO:TDI = 41:58:1 wt.%) and BCP/GO membranes in pervaporation dehydration of ethanol (4.4-70 wt.% water) at 22°C.

Transport parameter	Membrane / Water content in feed, wt.%	BCP	BCP/GO (0.3%)	BCP/GO (0.5%)	BCP/GO (0.7%)	BCP/GO (0.9%)
Separation factor (β)	4.4	64	63	66	72	50
	10	31	31	32	36	23
	30	13	14	15	16	9
	50	9	8	9	17	6
	70	12	11	20	34	7
Membrane selectivity (α)	4.4	65	64	67	74	51
	10	32	32	33	37	24
	30	13	14	15	16	9
	50	9	8	9	17	6
	70	12	11	20	34	7
PSI	4.4	2.8	3.3	4.2	5.5	3.4
	10	1.4	1.6	2.0	2.7	1.5
	30	0.6	0.7	0.9	1.2	0.6
	50	0.4	0.4	0.5	1.3	0.3
	70	0.6	0.6	1.3	2.9	0.5
